# Responses of Microbial Community Composition to Temperature Gradient and Carbon Steel Corrosion in Production Water of Petroleum Reservoir

**DOI:** 10.3389/fmicb.2017.02379

**Published:** 2017-12-05

**Authors:** Xiao-Xiao Li, Tao Yang, Serge M. Mbadinga, Jin-Feng Liu, Shi-Zhong Yang, Ji-Dong Gu, Bo-Zhong Mu

**Affiliations:** ^1^State Key Laboratory of Bioreactor Engineering and Institute of Applied Chemistry, East China University of Science and Technology, Shanghai, China; ^2^Shanghai Collaborative Innovation Center for Biomanufacturing Technology, Shanghai, China; ^3^School of Biological Sciences, The University of Hong Kong, Hong Kong, Hong Kong

**Keywords:** microbial induced corrosion, temperature gradient, biocorrosion, mesophilic, thermophilic and hyperthermophilic microorganisms, oil reservoir

## Abstract

Oil reservoir production systems are usually associated with a temperature gradient and oil production facilities frequently suffer from pipeline corrosion failures. Both bacteria and archaea potentially contribute to biocorrosion of the oil production equipment. Here the response of microbial populations from the petroleum reservoir to temperature gradient and corrosion of carbon steel coupons were investigated under laboratory condition. Carbon steel coupons were exposed to production water from a depth of 1809 m of Jiangsu petroleum reservoir (China) and incubated for periods of 160 and 300 days. The incubation temperatures were set at 37, 55, and 65°C to monitoring mesophilic, thermophilic and hyperthermophilic microorganisms associated with anaerobic carbon steel corrosion. The results showed that corrosion rate at 55°C (0.162 ± 0.013 mm year^-1^) and 37°C (0.138 ± 0.008 mm year^-1^) were higher than that at 65°C (0.105 ± 0.007 mm year^-1^), and a dense biofilm was observed on the surface of coupons under all biotic incubations. The microbial community analysis suggests a high frequency of bacterial taxa associated with families Porphyromonadaceae, Enterobacteriaceae, and Spirochaetaceae at all three temperatures. While the majority of known sulfate-reducing bacteria, in particular *Desulfotignum*, *Desulfobulbus* and *Desulfovibrio* spp., were predominantly observed at 37°C; *Desulfotomaculum* spp., *Thermotoga* spp. and *Thermanaeromonas* spp. as well as archaeal members closely related to *Thermococcus* and *Archaeoglobus* spp. were substantially enriched at 65°C. Hydrogenotrophic methanogens of the family Methanobacteriaceae were dominant at both 37 and 55°C; acetoclastic *Methanosaeta* spp. and methyltrophic *Methanolobus* spp. were enriched at 37°C. These observations show that temperature changes significantly alter the microbial community structure in production fluids and also affected the biocorrosion of carbon steel under anaerobic conditions.

## Introduction

Microorganisms in subsurface oil reservoirs and production system can lead to serious economic and safety problems, including reservoir souring and corrosion of the carbon-steel infrastructure. Water flooding has been a common and widely accepted method for increasing oil recovery from petroleum reservoirs (Ren et al., 2015). Distinct thermal characteristics of subsurface and surface environments can cause a considerable variation of the production fluid temperature in the oil production systems. The production fluids can provide mesophilic (20–40°C at surface of facilities and wellhead), thermophilic (> 45°C, middle part of wellbore) and hyperthermophilic (> 60°C, deep wellbore and reservoir) habitats (Song et al., 2017). Despite the extensive study of petroleum reservoir, information about the potential of microbially influenced corrosion (MIC) of pipeline under the prevailing environmental condition is still lacking.

**FIGURE 1:**
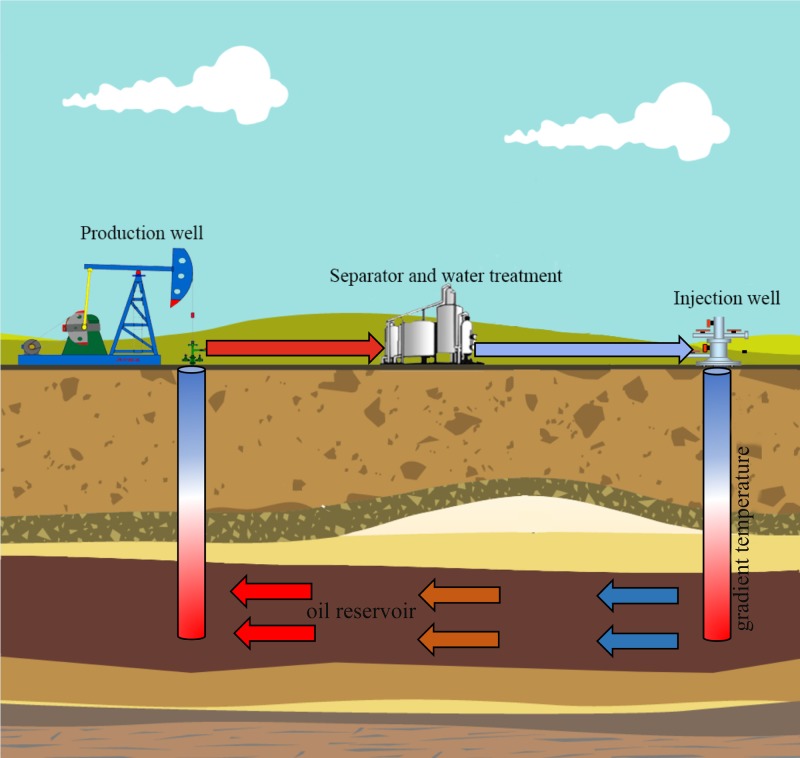
Schematic diagram of temperature gradient in cyclic production system in petroleum reservoir.

Jiangsu oil reservoir is known for its high rate of corrosion and reservoir souring, and the time span for pipeline corrosion failures have reduced to just 3–4 months from the initial about one and half a years due to the souring and accelerated corrosion in recent years. Furthermore, high corrosion and souring cause a loss of approximately 85 million RMB annually to the entire oilfield (Guan et al., 2014). The production water of petroleum reservoirs can be retrieved at the well bottom (subsurface) or the wellhead (surface). The *in situ* oil well bottom sampling (well bottom) is considered to be more accurate than wellhead fluids. However, the subsurface sampling (well bottom) requires advanced down-hole tools and production halts with a prohibitive expense. Therefore, wellhead sampling is still irreplaceable for engineering-oriented field evaluation and especially for the microbial monitoring during oil production. The Jiangsu oilfield experience corrosion problems also with the wellhead fluids (surface pipelines/facilities) that have lower temperature with a corrosion-monitoring program. In order to reduce the damaging effects of microbial growth and activity, the oilfield had been used broad-spectrum biocides or inhibitors historically. The oil well selected here due its exclusively corrosion in the surface portions of the oil transport system including equipment used in oil production, transportation and storage under conditions of lower temperature. In the cyclic oil production systems, the wellbore and treatment facilities often have a temperature gradient as shown in **Figure 1**. It is hard to know the actual corrosion rates and microbial compositions at various locations of an oil well pipeline. Therefore, accurate characterization of microbial communities responsible for corrosion and their metabolic potential at different temperature conditions become especially important for evaluating the pipelines safety and development of management strategies to prevent biocorrosion.

Microorganisms are involved and responsible for different mechanisms that lead to pipeline corrosion failures (Voordouw et al., 2016). Sulfate-reducing bacteria (SRB) are often considered as the principal agent for MIC and have been most thoroughly studied under anaerobic conditions and monitored routinely in the industry (Cetin and Aksu, 2009; Gu et al., 2011; Gu and Mitchell, 2013; Chen et al., 2014, 2015). SRB contribute to MIC by metabolizing the available electron donors (sulfate, thiosulfate) to produce hydrogen sulfide referred to as chemical MIC (CMIC) (Dinh et al., 2004; Enning et al., 2012; Venzlaff et al., 2013). The produced sulfide can react with carbon steel to form hydrogen, which is then also used by SRB. Some SRB are also capable of extracting electrons from metallic iron directly referred to as electrical MIC (EMIC) (Enning et al., 2012; Enning and Garrelfs, 2014). In addition, the acid-producing fermentative microorganisms and methanogens might indirectly increase corrosion through the production of organic acids or syntrophy with corrosive microorganisms (Gieg et al., 2014; Mand et al., 2014; Kip and Veen, 2015). The involvement of syntrophic metabolism in biocorrosion when a natural microbial community was grown with iron as the sole electron donor has been demonstrated (Usher et al., 2015). Furthermore, anaerobic biodegradation of fuel components coupled with sulfate respiration greatly contributed to the biocorrosion of carbon steel (Liang et al., 2016). Biological reactions, growth and activity of microbial community are greatly influenced by temperature. Previous studies also showed that temperature changes could alter the dominant anaerobic metabolic pathways and associated microbial populations (Conrad et al., 2009; Zeng et al., 2014).

The main objective of this study were to investigate the responses of microbial community to temperature in oil production system, and study the corrosion of carbon steel and identify the mesophilic and thermophilic microorganisms related to biocorrosion under simulated anaerobic conditions.

## Materials and Methods

### Description of Site and Samples

Production water was collected from Wei 9–18 oil producing well located in the Jiangsu oil field (Yangzhou, Jiangsu, China). Production water sampling was performed in November 2015. Physicochemical characteristics of the production fluid were described previously. There are also some microbiological data and related corrosion rates of others oil wells in Jiangsu oil reservoir (Li et al., 2017a). The procedure of sampling was similar to those reported previously. After initial flushing and then production water was collected directly from the production valve of the pipeline at the well head into sterile bottles to completely fullness before capping. The bottles were tightly sealed and immediately transported back to laboratory for treatment. The production water was obtained from a depth of 1809 m with an estimated temperature 79.6°C, and a corrosion rate was 0.031 mm year^-1^, which is provided by the Jiangsu Oilfield Company. The produced fluids in surface facilities and wellhead is 20–40°C, middle part of wellbore is above 45°C, and deep wellbore and reservoir above 60°C. We try to mimic the *in situ* temperatures (37, 55, and 65°C) to represent different part of the oil production systems. The corrosion rate (at the oil well temperature) was determined according to the weight loss method with the wells temperature in a week. Briefly, carbon steel coupons (20#) were immersed in 250 ml glass bottles filled with oil well production water. The production water was then used as both medium and inoculum source to investigate the response of microbial population to elevated temperature and the potential impact on the corrosion of carbon steel. Incubations were constructed under anaerobic conditions in the presence of carbon steel coupon. The surface finish of carbon steel coupons was as received. Microcosms were prepared by transferring 100 ml production water into 120-mL serum bottle containing one carbon steel coupon (50 mm × 10 mm × 1.5 mm) under a stream of positive N_2_ pressure (99.99% purity), and then sealed with butyl rubber stopper and aluminum crimp seal (Yang et al., 2016). The sampling of the culture was carry out using sterile and degassed syringes and needles. Sterile negative control microcosms were prepared with autoclaved production water. Incubations were maintained in the dark at 37, 55, and 65°C in triplicates, respectively.

### DNA Extraction and PCR Amplification

At the end of the incubation periods (160 and 300 days), the carbon steel coupons were removed from the serum bottles under positive pressure of N_2_ gas flow. 20 ml of active culture from one of the three replicates were withdrawn from the bottle, and then centrifuged at 12,000 rpm at 4°C for 10 min (Mbadinga et al., 2012; Pan et al., 2017) to collect the microbial cells. Total genomic DNA was extracted by using the AxyPrep^TM^ Bacterial Genomic DNA Miniprep Kit (Axygen Biosciences, Inc., Union City, CA, United States) according to the manufacturer’s instructions and stored at -20°C before further treatment. The universal primer sets of 8F (5′-AGAGTTTGATYMTGGCTCAG-3′)/805R (5′-GACTACCAGGGTATCTAATCC-3′) (Edwards et al., 1989; Turner et al., 1999; Savage et al., 2010) and 109F (5′-ACKGCTCAGTAACACGT-3′)/912R (5′-CTCCCCCGCCAATTCCTTTA-3′) (Großkopf et al., 1998; Nazina et al., 2006) were used for bacterial and archaeal 16S rRNA gene amplification, respectively. PCR conditions for bacterial 16S rRNA genes were as follows: an initial denaturation step at 95°C for 5 min, followed by 36 cycles of denaturation at 94°C for 30 s, 52°C for 40 s, 72°C for 60 s, and a final elongation step at 72°C for 10 min. PCR conditions for archaeal 16S rRNA genes were as follows: an initial denaturation step at 95°C for 5 min, followed by 32 cycles of denaturation at 94°C for 45 s, 60°C for 40 s, 72°C for 60 s, and a final elongation step at 72°C for 10 min.

### Construction of 16S rRNA Gene Libraries

The PCR-amplified products were purified using Gel Extraction kit (Axygen Biosciences, United States). The purified DNA fragments were cloned into *Escherichia coli* using the pMD^®^19-T simple vector (Takara Biomedical Technology, Japan) according to manufacturer′s protocol. White clones were randomly picked and inoculated into 0.8 mL of ampicillin-added luria broth (LB) medium for 24 h at 37°C. The positive clones were analyzed by PCR amplification with the vector specific primer set of M13–47 (5′-CGCCAGGGTTTTCCCAGTCACGAC -3′) and RV-M (5′-GAGCGGATAACAATTTCACACAGG) to check correct insert as described previously (Liu et al., 2017). The clone number of each sample was shown in **Table 1**. The reactions were carried out as follows: initial denaturation of DNA at 95°C for 5 min, 20 cycles of denaturation at 95°C for 1 min, primer annealing at 52°C for 45 s, and extension at 72°C for 1 min, a final extension at 72°C for 10 min. Sequencing of the positive clones was accomplished on an ABI 377 automated sequencer. Sequences were first aligned with MAFFT version 7 (Katoh and Standley, 2013), then trimmed manually to remove vector using MEGA6.0 (Tamura et al., 2013). Chimeric sequences were checked and discarded by Bellerophon (Huber et al., 2004). Representative sequences of OTU (97% similarity for 16S rRNA) were determined using CD-HIT v4.6 (Fu et al., 2012). Representative Sequence of each OTU was compared to the GenBank Database to match the most similar sequence by using BLAST.

**Table 1:** Numbers of sequenced clones in each stage of incubation sample, Alpha diversity and GenBank accession numbers.

Type	Sample	No. of	No. of OTUs	Shannon diversity	Simpson (inverse)	S_Chao1_	S_ACE_	Accession
		sequences		index	index			numbers
Bacteria	37C-160D	120	21	2.25	6.01	32	40	MF470551-MF470670
	37C-300D	98	17	2.42	8.89	24	23	MF470795-MF470892
	55C-160D	124	6	1.36	3.36	7	7	MF470671-MF470794
	55C-300D	92	9	1.71	4.64	10	15	MF470379-MF-470470
	65C-160D	133	6	1.45	3.80	6	6	MF470893-MF471025
	65C-300D	80	12	2.02	5.94	13	15	MF470471-MF470550
Archaea	37C-160D	99	6	0.53	1.29	9	11	MF471026-MF471124
	37C-300D	107	7	1.27	2.86	8	9	MF465574-MF465680
	55C-160D	110	1	0	1	1	1	MF465219-MF465328
	55C-300D	113	1	0	1	1	1	MF465329-MF465441
	65C-160D	92	3	0.95	2.36	3	3	MF465442-MF465533
	65C-300D	95	3	0.79	2.05	3	3	MF465681-MF465775


### Corrosion Evaluation

The corrosion rate was determined after 28 days of incubation from the metal weight loss method. Corrosion rate = (*K* × *W*) / (*A* × *T* × *D*), where *K*, constant (3650); *W*, mass loss (mg); *A*, area of carbon steel coupon (cm^2^); *D*, density of the coupon (g cm^-3^); *T*, the incubation time (days). Carbon steel coupons, after removal from the serum bottle, were fixed in a phosphate buffer solution containing 2.5% glutaraldehyde for 4 h, and then dehydrated with an ethanol series. The biofilm and corrosion products of selected coupons from each microcosm were analyzed with a scanning electron microscope (SEM) coupled with an energy-dispersive X-ray spectrometer (EDS) (Cheng et al., 2016). Dissolved sulfide in bottles were determined by using the methylene blue method (Tanner, 1989).

### Statistical Analyses

Based on OTU screening, Shannon index, reciprocal Simpson index and the non-parametric richness estimators ACE and Chao 1 were calculated using Estimates (Colwell et al., 2012). Bubble plots were made using the software R with the ggplot2 (Wickham, 2009) and reshape2 (Wickham, 2012) packages. Ternary plots were also created with ggplot2 and ggtern (Hamilton, 2016) in R. Statistical differences between the samples in terms of microbial communities^,^ composition and principal components analysis (PCA) analysis of the microbial community pattern were carried out with STAMP software package (Parks et al., 2014). Differences in taxa relative abundance between samples were assessed with the extend error bar method in STAMP using the two-sided Fisher’s exact test (Rivals et al., 2006).

## Results

### Corrosion and Corrosion Rates

**FIGURE 2:**
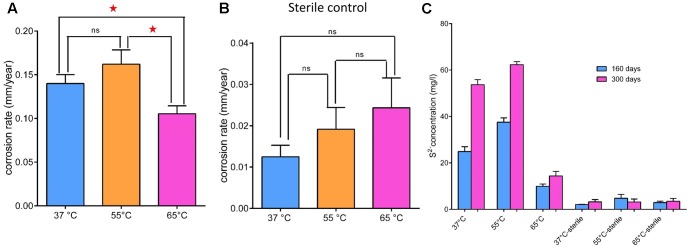
General corrosion rates **(A,B)** and dissolved sulfide **(C)** in the aqueous phase of microcosms under 37°C (blue bar), 55°C (orange bar) and 65°C (pink bar) after 28 days incubation. **(A)** Production water and carbon steel incubated at above temperatures; **(B)** corresponding sterile controls; **(C)** dissolved sulfide in the aqueous phase of microcosms after 160 (blue bar) and 300 days (pink bar) incubation. Asterisks indicate that differences among the means represented by the columns are statistically significant (*p* < 0.05). ns, no significant difference.

**FIGURE 3:**
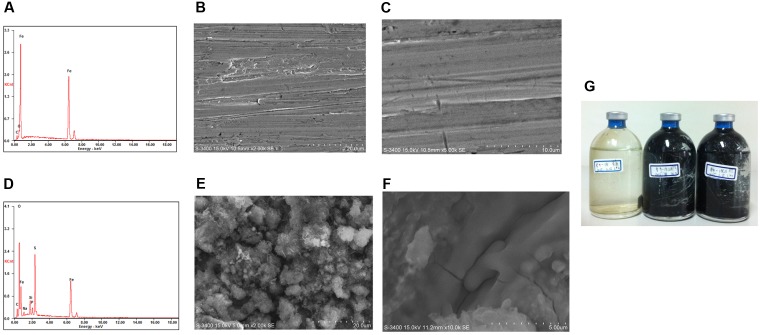
Energy-dispersive X-ray spectrometer (EDS) and scanning electron microscope (SEM) images of surface of carbon steel coupon before exposure **(A–C)** and an example after exposure **(D–F)** in production water environment after 28 days incubation; picture of incubations after 300 days **(G)**.

The average corrosion rate of the carbon steel coupons in the production water microcosms under the three temperatures (0.105 ± 0.007–0.162 ± 0.013 mm year^-1^) (**Figure 2A**) was much higher than that measured in the corresponding coupons in the sterile controls (0.013 ± 0.002–0.024 ± 0.006 mm year^-1^) by an order of magnitude (**Figure 2B**). The corrosion rate of carbon steel coupons detected in the microcosm at 65°C was significantly different from microcosms incubated under 55°C (*p* = 0.003) and 37°C (*p* = 0.01). However, the corrosion rate in the 37°C microcosms was not significantly different from the 55°C microcosms (*p* = 0.11). The corrosion rates of the sterile controls had no significantly different from each other. During the long term incubations (160 and 300 days), the pH increased from 8.0 of the initial production water to 8.7–9.6. The sulfate concentration decreased from 182 mg l^-1^ in the production water to 76–94 mg l^-1^ in the biotic incubations. The dissolved sulfide (9.95 ± 0.79–62.30 ± 1.09 mg l^-1^) in the microcosms was much higher than those in the sterile controls (2.07 ± 0.05–4.76 ± 1.37 mg l^-1^) (**Figure 2C**). After 28 days of exposure to production water, a significant blackening was observed in the incubations compared to the sterile controls (**Figure 3**). Energy dispersive spectroscopy revealed that the presence of sodium, silicon, sulfur, and phosphorus elements on the surface of carbon steel coupons over time of incubation (**Figures 3A,D**). SEM examination showed that microbial cells embedded in the corrosion deposits were evident on the surface of carbon steel coupons.

### Richness and Diversity of the Communities

Overall, 1263 of 16S rRNA gene sequences were used for microbial community analysis, and the clone number of each sample is shown in **Table 1**. The bacterial diversity and OTU number of each microcosm were higher than archaeal library. Shannon values were observed to be highest at 37°C (2.25–2.42 for bacteria, 0.53–1.27 for archaea), lower at 65°C (1.45–2.02 for bacteria, 0.79–0.95 for archaea) and lowest at 55°C (1.36–1.71 for bacteria, 0 for archaea). 37C-160D had a higher Chao 1 and ACE index. The inverse index values also revealed the same tread in diversity succession compared with Shannon index.

### Microbial Community Composition

**FIGURE 4:**
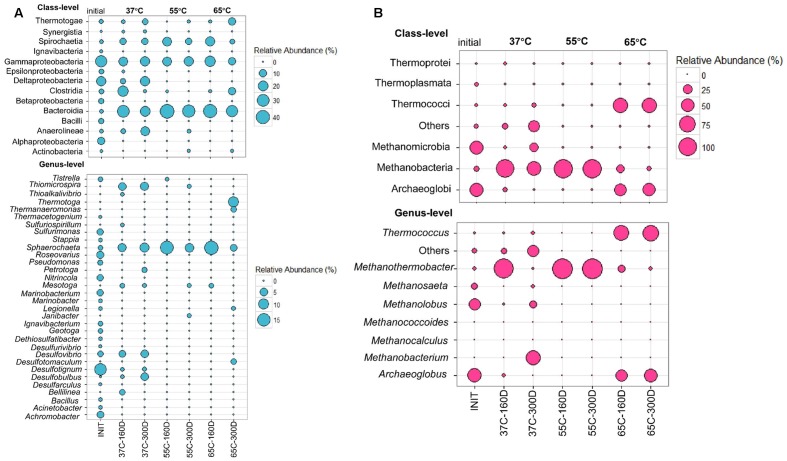
Bubble plots showing the relative abundance >0.5% of bacterial **(A)** and archaeal communities **(B)** in at least one sample at the class and genera levels. Abundance less than 0.5% of the total composition in all libraries are defined as “others.” INIT represents initial production water of petroleum reservoir, for details see Li et al. (2017a); 37C-160D means microcosms incubated at 37°C after 160 days; 37C-300D means microcosms incubated at 37°C after 300 days, and so on.

Bacterial community composition in the inoculum sample library was dominated by Gamma- (28.66%), Delta- (18.37%) and Alphaproteobacteria (9.81%). Each microcosm was mainly composed of Bacteroidia (20.41–44.35%), Gammaproteobacteria (10–21.8%) and Spirochaetia (3.75–18.8%) at all temperatures from 37 to 65°C (**Figure 4A**). The 37C-160D and 37C-300D samples were incubated at 37°C for 160 and 300 days. Class Synergistia made up 0.83 and 1.02% in 37C-160D and 37C-300D, respectively. Two samples at 37°C also contained Deltaproteobacteria, accounting for 5 and 18% of sequences in the samples of 37C-160D and 37C-300D, respectively. The sequences affiliated with Deltaproteobacteria were primarily consisted of three genera, *Desulfovibrio*, *Desulfotignum*, and *Desulfobulbus.* They seemed to be mesophilic rather than thermophilic as they were only detected at 37°C, but not detected at 55 or 65°C. Sequences affiliated with *Desulfotomaculum* (2.5%), *Thermotogae* (10%) and *Thermanaeromonas* (2.5%) were identified in 65C-300D, indicating they were more favored at hyper-thermophilic conditions. Meanwhile, Anaerolineae was also largely present in samples at 37°C, but less frequent in the others samples. Within the archaeal community, Methanomicrobia (43.56%) and Archaeoglobi (46.62%) predominated in the inoculum archaeal community (**Figure 4B**). Thermococci were highly enriched in 65C-160D (54.35%) and 65C-300D (57.89%). Methanobacteria (49.53–100%) shared a high portion in samples incubated at 37 and 55°C.

### Comparison of Bacterial and Archaeal Composition among the Microcosms

**FIGURE 5:**
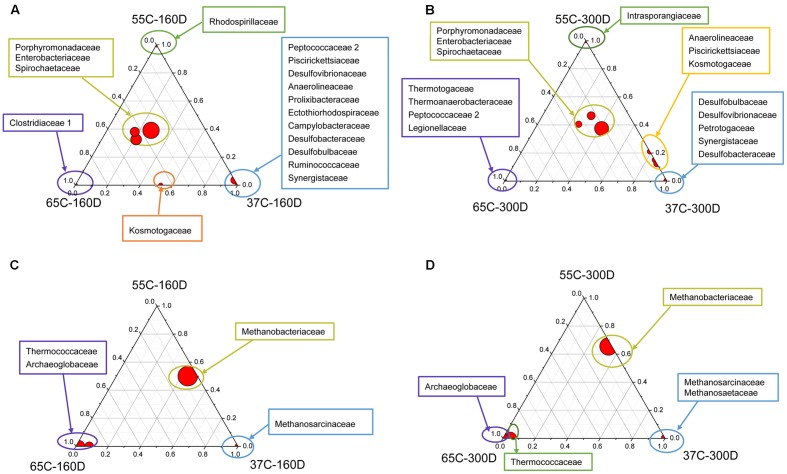
Distribution of taxonomic groups in different temperature culture microcosms with 160 **(A,C)** and 300 **(B,D)** days incubation. The percentage of bacterial **(A,B)** and archaeal **(C,D)** families associated with each microcosm is visualized in ternary plots. The position in triangle indicates the relative abundance of each taxon among the three cultured microcosms, the size of the circle represents the relative abundance of taxa.

The ternary plots of main families showed that Porphyromonadaceae, Enterobacteriaceae and Spirochaetaceae were the most abundant bacterial families detected in all samples after 160 and 300 days of incubation (**Figures 5A,B**), while Thermotogaceae and Thermoanaerobacteraceae were primarily identified in 65C-300D. Bacterial communities in 37C-160D were characterized by many unique families, detected in neither 55C-160D nor 65C-160D. Methanobacteriaceae was the dominant archaeal family in the samples incubated at 37 and 55°C, but less abundant at 65°C (**Figures 5C,D**). Methanosarcinaceae and Methanosaetaceae were only found in samples at 37°C. Thermococcaceae and Archaeoglobaceae were primarily detected in 65C-160D and 65C-300D microcosms, they were rarely detected at 37 and 55°C.

### Responses of Microbial Community to Temperature Gradient

**FIGURE 6:**
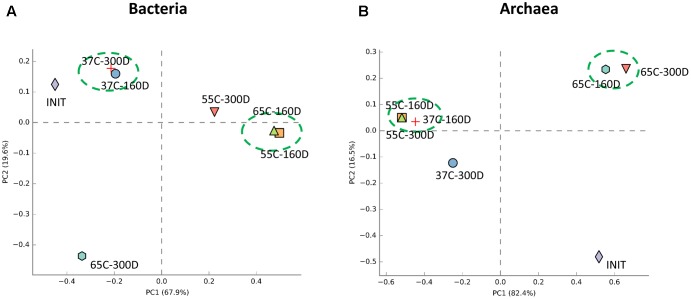
Principal components analysis (PCA) analysis of the bacterial **(A)** and archaeal **(B)** communities structure in the different temperature culture microcosms (six samples) compared to the inoculum sample. PCA analysis was carried with STAMP software package using ANOVA as statistical test (*p*-value < 0.05) and Turkey-Kramer as *post hoc* test.

**FIGURE 7:**
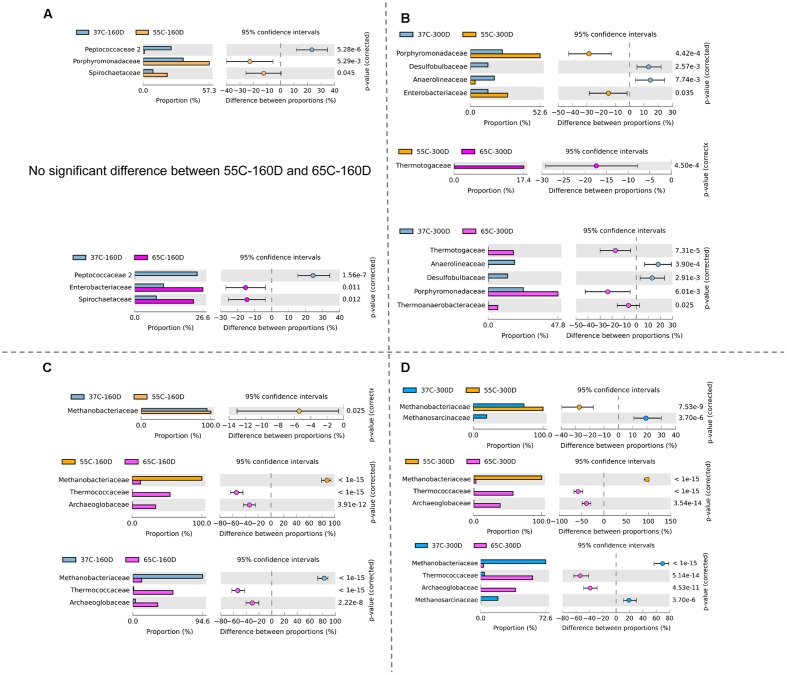
Bacterial **(A,B)** and archaeal **(C,D)** communities differences at the family level as revealed by the extended error bar method in STAMP using the two-sided Fisher’s exact test.

Principal components analysis analysis was used to visualize the structure of microbial community among samples (**Figure 6**). For the bacterial community, 37C-160D and 37C-300D, 55C-160D and 65C-160D clustered closely and were distinct different from the initial 55C-300D and 65C-300D. In terms of archaeal community, archaeal communities of 65C-160D and 65C-300D clustered closely together and appeared dissimilar from the initial microcosms and incubations at 37 and 55°C. The microbial community differences at family level were then compared using the extended error bar method in STAMP (Parks et al., 2014). The analysis showed an enrichment of the bacterial family Spirochaetaceae (*p* < 0.05) in the 55C-160D and 65C-160D compared to 37C-160D. Porphyromonadaceae (*p* < 0.01) also enriched in 55C-160D. Peptococcaceae 2 (*p* < 0.001) was substantially enriched in 37C-160D, while Enterobacteriaceae (*p* < 0.05) was slightly enriched in the 65C-160D. No significant difference was observed between the bacterial families between 55C-160D and 65C-160D. After a longer time of incubation, there was an over-representation of Desulfobulbaceae and Anaerolineaceae in 37C-300D (*p* < 0.01). Thermotogaceae (*p* < 0.001), Porphyromonadaceae (*p* < 0.01) and Thermoanaerobacteraceae (*p* < 0.05) were significantly abundant in 65C-300D microcosms. The family Porphyromonadaceae (*p* < 0.001) and Enterobacteriaceae (*p* < 0.05) presented a significant enrichment in 55C-300D (**Figures 7A,B**). Regarding the archaeal community, family Methanobacteriaceae (*p* < 0.05) was significantly enriched in 55C-160D and 55C-300D and Thermococcaceae (*p* < 0.001) and Archaeoglobaceae (*p* < 0.001) were more abundant in 65C-160D and 65C-300D. In addition, there was an enrichment of the family Methanosarcinaceae (*p* < 0.001) in 37C-300D (**Figures 7C,D**).

## Discussion

It is well known that corrosion of carbon-steel pipelines is a complex process involving multiple mechanisms influenced to varying degrees by the activity of microorganisms (Davidova et al., 2012). In this study, the mixed production water was collected from oil wellhead, it is reasonable to assume that the composition of the microbial communities at all three temperatures would be similar to each other after a short time incubation. On the other hand, the biofilms formation during the incubation may decrease the corrosion rate. Therefore, we measured the corrosion rates after 28 days. It has been showed that the general corrosion and pitting corrosion of carbon steel were positively correlated with sulfate loss in the incubations of marine environment (Liang et al., 2016). Temperature changes in short period incubation might not alter the composition of microbial community in production water significantly. Moreover, because of the pipeline corrosion failures time span at specific conditions, we are more interested in identifying the mesophilic and thermophilic microorganisms related to biocorrosion under a long-term incubations. Herein, we mainly sought to assess the potential biocorrosion via microbial community analysis of the long-term incubations in order to gain insight on the metabolic activities that might sustain these microorganisms.

### Methanogens Are Potential Contributors to MIC alongside SRB under Mesophilic Conditions

Sulfate-reducing bacteria have long been “blamed” as the main or even the sole culprits of MIC (Geissler et al., 2015; Voordouw et al., 2016). Here, some representatives of SRB within Deltaproteobacteria such as *Desulfovibrio*, *Desulfotignum*, and *Desulfobulbus* genera were only detected at 37°C (37C-160D and 37C-300D) (**Figure 4**). Obviously, they were more favored at mesophilic conditions, as they were non-detectable at 55 or 65°C. Besides, in the initial wellhead fluid, we also observed a high proportion of Deltaproteobacteria. Although they often occurred in production water of high temperature oil reservoirs (Guan et al., 2013; Li et al., 2016; Tian et al., 2017), it is also possible that they might come from cool portions of the wellbores not the deep oil reservoirs. And it has been reported that the wellhead samples of high-temperature, low-permeability petroleum reservoirs could represent the communities inhabiting the wellbores, instead of the reservoirs (Song et al., 2017). *Desulfovibrio* species are metabolically diverse and often coupling hydrogen or volatile fatty acid consumption with sulfate, iron, or nitrate reduction (Bryant et al., 1977). Molecular characterization of corrosive biofilms from offshore pipelines revealed that *Desulfovibrio* species dominated the microbial communities (Vigneron et al., 2016). Furthermore, several members of *Desulfovibrio* have been demonstrated to exacerbate corrosion of iron through EMIC occurring on metal surface (Enning and Garrelfs, 2014). *Desulfotignum*-species, known as mesophilic SRB, could use a large variety of substrates, ranging from simple organic compounds and fatty acids to aromatic compounds as electron donors, and sulfate, thiosulfate and sulfite were used as electron donors (Kuever et al., 2001). Furthermore, it has been reported that many sulfate-reducers can oxidize saturated hydrocarbons (Mbadinga et al., 2011), and the syntrophic hydrocarbon metabolism coupled with SRB might be involved in catalyzing the pitting corrosion of carbon steel (Liang et al., 2017).

Methane and hydrogen production were observed at incubations containing carbon steel coupons and the abiotic controls, respectively. Previous study showed that carbon steel, not oil constituent organics compounds, was the predominant electron donor for acetate and methane formation under relatively low temperature (30°C) mesophilic Canadian oil fields (Mand et al., 2015). Hydrogenotrophic methanogen of the *Methanothermobacter* genus was the most prevalent and observed at all temperatures, but *Methanosaeta*, *Methanolobus* and *Methanobacterium* genera were only found at 37°C. It has been showed that electrons could be transferred directly from metallic coupons to *Methanosaeta* to reduce carbon dioxide to methane, implying that *Methanosaeta* could also play a role in biocorrosion via cathodic depolarization (Rotaru et al., 2014). In addition, *Methanosaeta* contribute indirectly to metal corrosion by using metabolic products produced by other potentially corrosive microorganisms such as SRB and acid-producing bacteria (Mand et al., 2014; Ozuolmez et al., 2015). Genus *Methanothermobacter* constituted the majority of the sequences in the incubation at 37°C after a period of 160 days. Interestingly, *Methanobacterium* turned to be the dominant microorganisms in the microcosms after 300 days of incubation (**Figure 4B**). *Methanobacterium* spp. are hydrogen scavengers and have been frequently detected at corrosive biofilms (Dinh et al., 2004; Vigneron et al., 2016).

### Biocorrosion Mainly Associated with Methanogenic Archaea under Thermophilic Environments

Hydrogenotrophic methanogens closely related to *Methanothermobacter* spp. were the most encountered archaeal taxa at 55°C. They are also commonly isolated from petroleum fluids and are known to make up a significantly portion of the microbial communities in such systems (Mayumi et al., 2011; Nakamura et al., 2013). It has been reported at temperatures higher than about 40°C, methanogenesis changed from a mixture of acetoclastic and hydrogenotrophic methanogenesis to exclusively hydrogenotrophic methanogenesis in anoxic rice field soils (Conrad et al., 2009). There is in consistency with our results here. There were acetoclastic and hydrogenotrophic methanogens at 37°C. While, at 55°C, All methanogens belonged to hydrogenotrophic methanogens (**Figure 4B**). Other studies on reservoir fluids also suggests dominance of hydrogenotrophic methanogenesis coupled to acetate oxidation at temperature above 50°C (Dolfing et al., 2008; Mayumi et al., 2011). These all suggests that temperature defined the structure and the function of the methanogenic community in anaerobic reservoir fluids. In terms of bacterial communities, families Porphyromonadaceae, Enterobacteriaceae and Spirochaetaceae were the most frequently detected (**Figure 5**) at 55°C. Information of these group related to corrosion is scarce, except a study revealed that members of the family Enterobacteriaceae may be involved in biocorrosion of metallic material in marine conditions (Bermont-Bouis et al., 2007). Other studies also recognized the high abundance of Enterobacteriaceae in liquid samples or laboratory biofilms that mimic the pipeline environment (Zhu et al., 2003; Jan-Roblero et al., 2004; Neria-González et al., 2006). Studies on corrosion by pure methanogenic cultures concluded that an increase in methanogen population correlated well with the corrosion rates (Daniels et al., 1987). Furthermore, methanogenic archaea have been linked to elemental iron oxidation and corrosion (Dinh et al., 2004; Uchiyama et al., 2010). It has been reported that microbial methane production associated with carbon steel corrosion in a Nigerian oil field (Mand et al., 2015). Moreover, a strong correlation between the methane productions and corrosion rate of the mild steel coupons in the samples from transporting pipes were also observed (Okoro et al., 2016). Presumably, methanogenic archaea may play a major role in the corrosion of carbon steel.

### Thiosulfate-Reducing Microorganisms Implicated with Biocorrosion of Pipelines under Hyperthermophilic Conditions

Biocorrosion mitigation efforts in oil fields are often targeted at the mesophilic SRB, and it is less common to monitor and recognize other H_2_S-producing microorganisms that reduce sulfur compound like thiosulfate other than sulfate (Lenhart et al., 2014). Recently, thiosulfate-reducing microorganisms have been implicated in the biocorrosion of a hot petroleum production facility by using both culture-dependent and culture-independent methods (Liang et al., 2014). Bacterial Taxa closely affiliated to genera *Desulfotomaculum*, *Thermanaeromonas* and *Thermotoga* were enriched in the microcosms at 65°C, and hyperthermophilic *Thermococcus* spp. and *Archaeoglobus* spp. dominated the archaeal communities (**Figure 4B**), and they are deemed to the typical residents in the deep subsurface oil reservoirs (Stetter et al., 1993; Orphan et al., 2000). Within Firmicutes, *Desulfotomaculum* spp. were frequently occurred in subsurface environments (Li et al., 2017b). *Desulfotomaculum thermocisternum*, a representative of this genus, isolated from a hot North Sea oil reservoir can use hydrogen and a number of organic compounds, such as lactate, ethanol and carboxylic acids in the presence of sulfate as electron acceptor (Nilsen et al., 1996). *Thermanaeromonas* spp. are known as strictly anaerobic and could use thiosulfate as electron acceptor (Geissler et al., 2015). Among archaeal community, several members of the genus *Thermotoga, Thermotoga neapolitana* and *Thermotoga maritima*, isolated from an oil well, could also reduce thiosulfate to sulfide (Ravot et al., 1995). *Thermococcus* strain PK can reduce elemental sulfur and producing highly corrosive H_2_S. Furthermore, if sulfur is not available, strain PK can grow by iron reduction or slowly by manganese reduction (Davidova et al., 2012). *Archaeoglobus* spp. with close affinity to *Archaeoglobus fulgidus* can reduce both sulfate and thiosulfate (Stetter et al., 1987). Therefore, thiosulfate-reducing microorganisms would play an important role in sulfur cycling and biocorrosion of pipelines under high temperature conditions.

## Conclusion

Temperatures had a significant impact on the microbial community composition and structure of oilfield system. With an increase of temperature, the dominance of taxonomical groups of microorganisms changed accordingly. Mesophilic SRB, acetoclastic and hydrogenotrophic methanogens were observed at 37°C compared to exclusively hydrogenotrophic methanogens at 55°C. Methanogens may contribute to biocorrosion by a syntrophy mechanism with SRB under mesophilic conditions, but the functional roles of three shared and dominant bacterial group Porphyromonadaceae, Enterobacteriaceae and Spirochaetaceae remains unclear. At higher temperature, hyperthermophilic sulfidogenic microorganisms, especially thiosulfate reducers, may involve in MIC. Our result expand current knowledge of the specific microorganisms in production fluids at different temperatures and provided a clearer picture of microbial risks in oil production system.

## Author Contributions

B-ZM and J-DG designed the experiments, X-XL conducted the experiments, X-XL and TY carried out the microbial analysis. J-FL, S-ZY and SM gave suggestions for the experiments and results analysis. X-XL prepared the manuscript with contribution from all co-authors.

## Conflict of Interest Statement

The authors declare that the research was conducted in the absence of any commercial or financial relationships that could be construed as a potential conflict of interest.
